# Supplementary material for: Senescent Secretome of Blind Mole Rat Spalax Inhibits Malignant Behavior of Human Breast Cancer Cells Triggering Bystander Senescence and Targeting Inflammatory Response
Source: Int J Mol Sci. 2023 Mar 7;24(6):5132. doi: 10.3390/ijms24065132 (PMC10049022; doi:10.3390/ijms24065132)
Supplement: Supplementary file 1 [file ijms-24-05132-s001.zip › ijms-2236675-supplementary.pdf]

Supplementary Materials

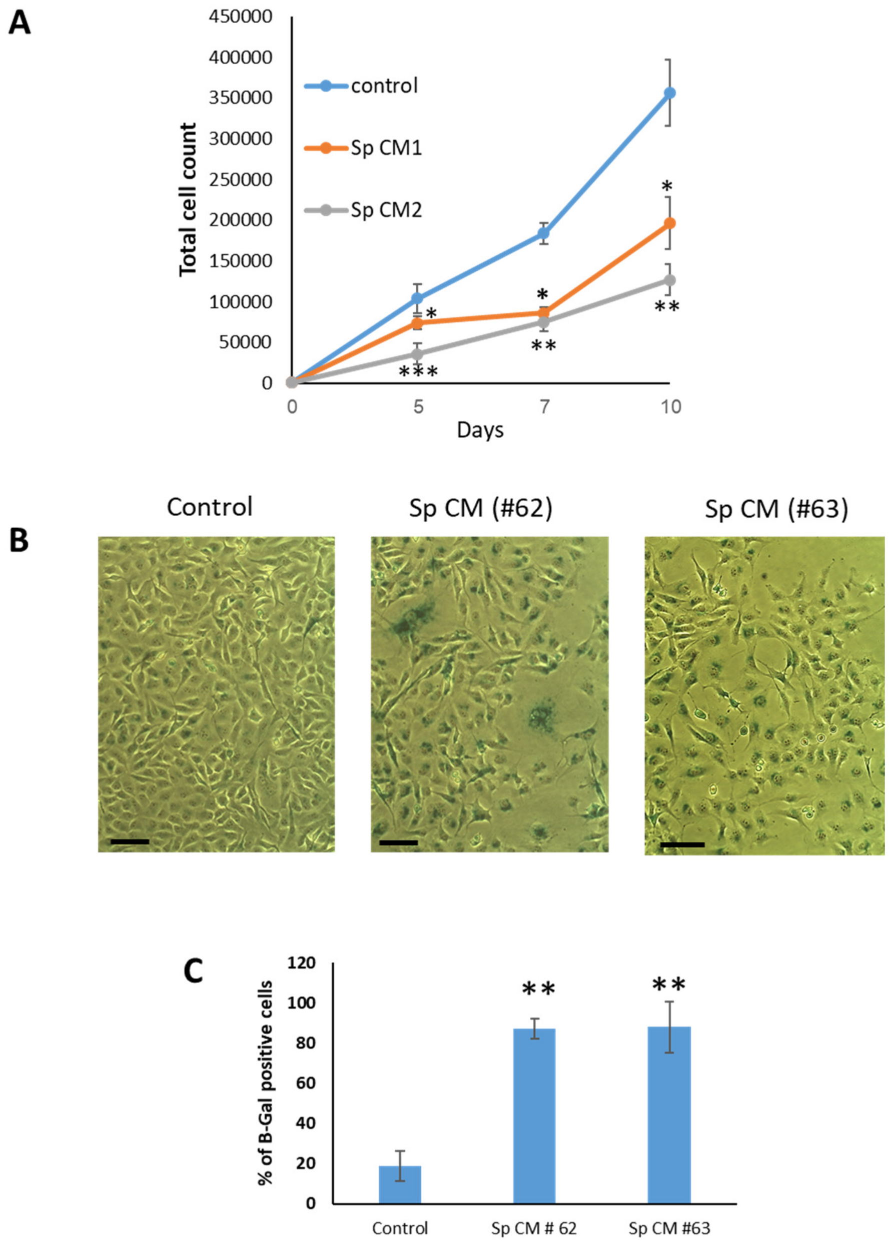

Figure S1. Influence of long-term cultivation of MDA-MB-231 cells with senescent *Spalax* secretomes on the level of proliferation and maintenance of senescence phenotype. (A) $5{\times}10^3$ cells were plated in 12-well plates under *Spalax* CMs (1:1 with fresh medium) or untreated, and counted after 5, 7 and 10 days. CM was replaced after 5 days of treatment with the same CM in a ratio of 1:1 with fresh nutrient media. CM #62 and #63 are media collected from cultures of late passage *Spalax* fibroblasts isolated from independent individuals (biological repeats). Values represent the average of at least two independent experiments in triplicate. Data presented as mean ± SD. * $p < 0.05$; **$p < 0.01$; *** $p < 0.001$ differences between control (untreated cells) and cells treated with *Spalax* CM (B) SA-β-Gal staining representative images after 10-days treatment of MDA-MB-231 cells with *Spalax* CMs. Bars, 100µm (C) Percentage of SA-β-Gal-positive cells calculated from at least 300 cells in four independent fields for each biological repeat (n = 2) in triplicate (*Spalax* CMs were collected from senescent cells of two independent individuals. ** $p < 0.01$ differences between control (untreated) and treated with *Spalax* CM.

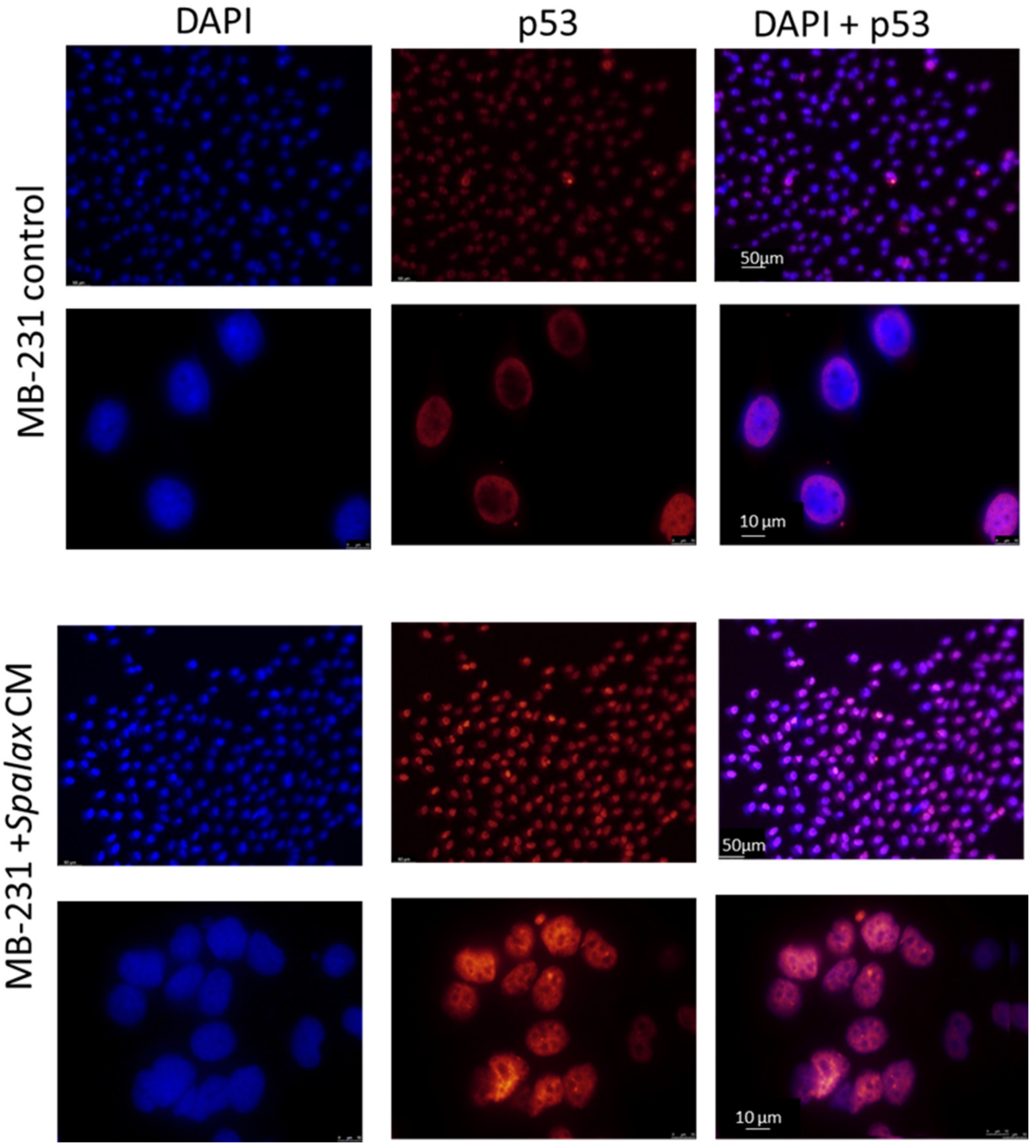

Figure S2. MDA-MB-231 cells were plated on coverslips at 40×10^3 cell/well in 6-well plates and treated with Spalax CM or untreated. After 4 days, the cells were washed with PBS, fixed, and permeabilized as described in Materials and Methods. For p53 visualization (1026-1) p53 RabMAb® (primary Ab) (Abcam, Cambridge, United Kingdom) and anti-rabbit alexa fluor 647 (ThermoFisher Scientific, Wohlen, Switzerland) were used, thereafter coverslips were counterstained with DAPI. Representative images of MDA-MB-231 untreated and treated with *Spalax* CM are presented.

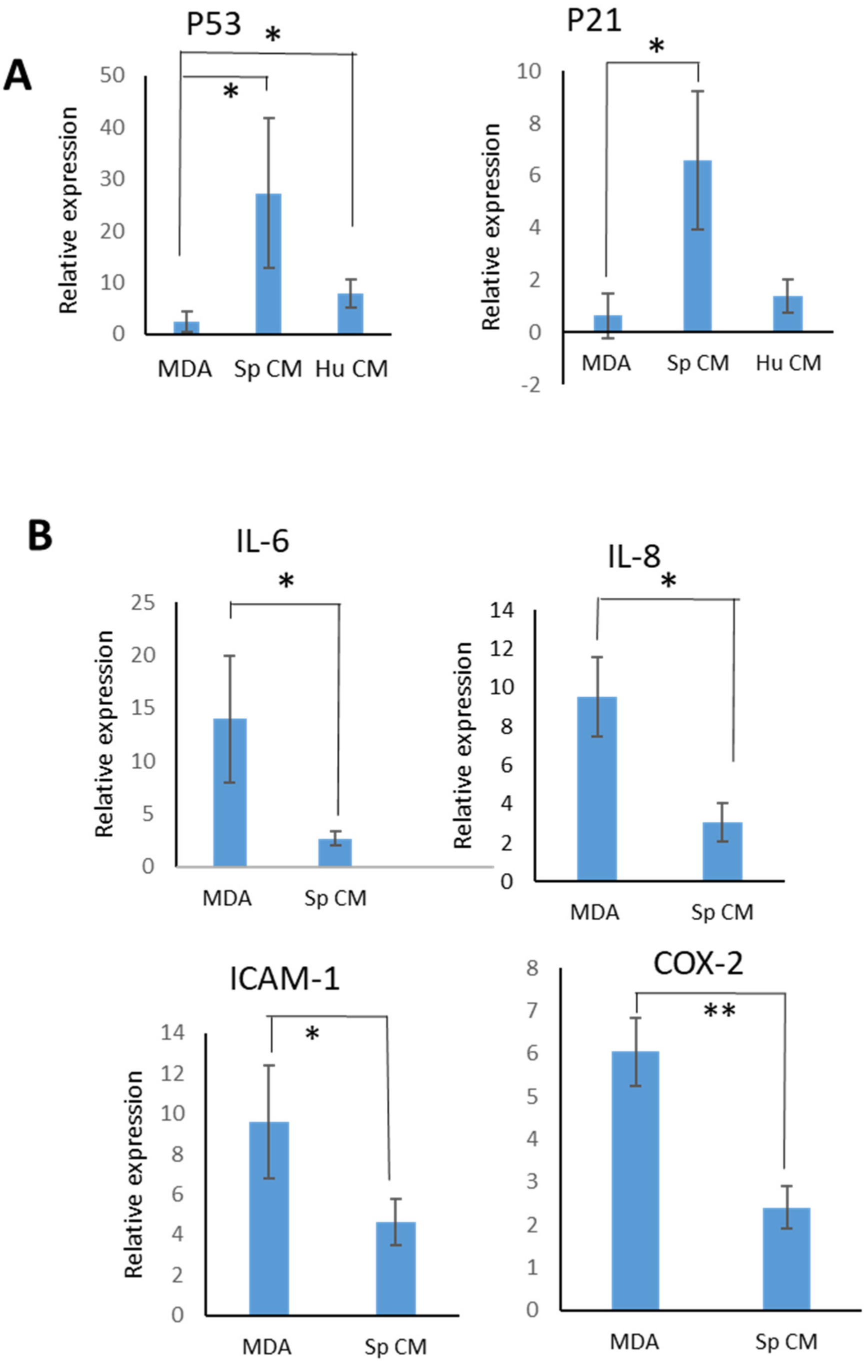

Figure S3. (A) Effects of long-term exposure of MDA-MB-231 cells with *Spalax* and human CMs on P53/P21 gene expressions**.** MDA-MB231 were incubated with CM from senescent *Spalax* and human fibroblasts for 10 days or untreated, with replacement medium after 5 days (1:1 with fresh medium). (B) The expression of SASP factors in MDA-MB-231 cells after exposure to Spalax CM for 10 days, compared to control (non-treated cells). The mRNA expression rates were quantified by using qRT-PCR. Data presented as mean ± SD. * $p < 0.05$; ** $p < 0.01$; differences between control (untreated) and treated with *Spalax* CM.

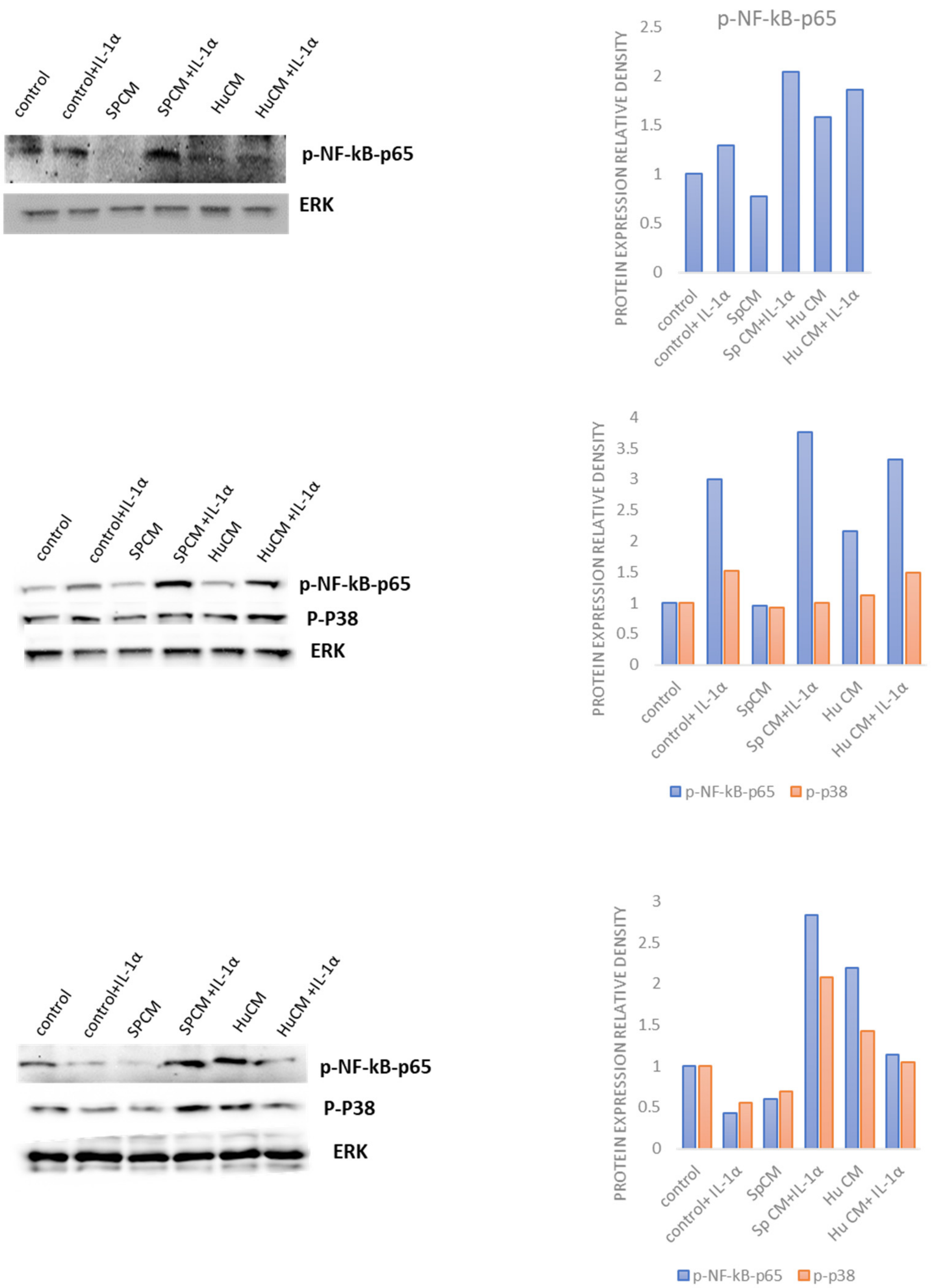

Figure S4. Biological repeats of western blot analysis presented in Figure 6C,D. WB demonstrating the phosphorylation of p65 (Ser536), and p-38 in untreated MDA-MB231, treated with recombinant IL-1α (control + IL1α), *Spalax* CM and human CM with or without IL1α; with corresponding densitometry quantification.

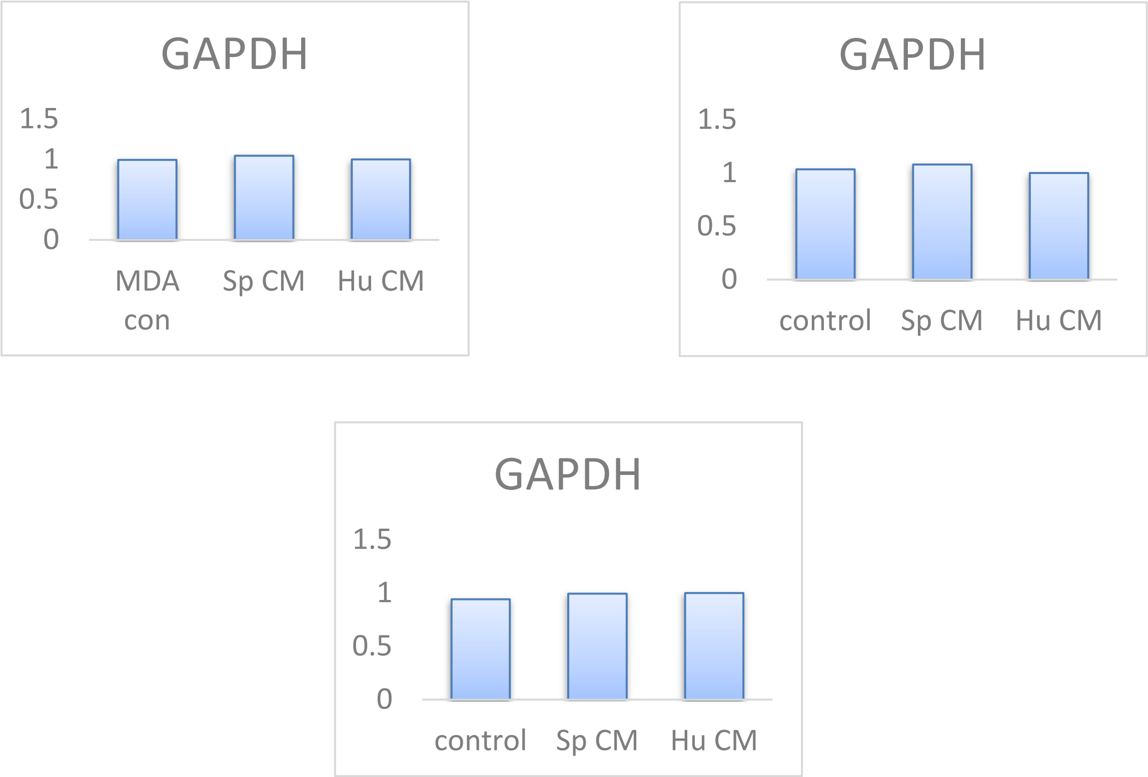

Figure S5. The mRNA expression of GAPDH in MDA-MB231 cells under exposure to *Spalax* or human CMs.

Table S1. List of antibodies.

Antigen Name	Dilution	Origin	Company
Primary antibodies Western blot analysis			
Anti- phospho-NF-kB p65 (Ser536)	1:1000	Rabbit	Cell signaling
Anti -IL-1alpha -FITC	1:1000	Rabbit	Avivasysbio
Anti-phospho-p38	1:1000	Rabbit	Abcam
Anti-actin	1:300	Goat	Santa Cruz
Antibodies for fluorescent microscopy			
Anti-gamma H2AX (phospho S139)	1:700	Rabbit	Abcam
Anti -IL-1alpha -FITC	1:1000	Rabbit	Avivasysbio
Anti- NF-kB p65	1:1000	Rabbit	Cell signaling
Secondary antibodies			
Anti-mouse CY3	1:300	Donkey	Jackson ImmunoResearch Lab. Inc.
Anti-rabbit CY2	1:300	Donkey	Jackson ImmunoResearch Lab. Inc.
Anti-rabbit Alexa 647	1:300	Goat	Abcam
Anti-rabbit Alexa 488	1:300	Goat	Abcam
Anti-rabbit HRP	1:10000	Goat	Jackson ImmunoResearch Lab. Inc.
Anti-goat HRP	1:10000	Donkey	Jackson ImmunoResearch Lab. Inc.

Table S2. Primers for RT-qPCR used in the studies.

**Primers**	**Forward**	**Reverse**
**Human primers**		
COX-2	TTTTGGTGGAGAAGTGGGTTTT	CCCTTCACGTTATTGCAGATGA
IL-6	TGTAGCCGCCCCACACA	CCGTCGAGGATGTACCGAAT
P53	CCGAGTGGAAGGAAATTTGC	TAGGGCACCACCACACTATGTC
P21	GCGGCAGACCAGCATGAC	GCGGATTAGGGCTTCCTCTT
HPRT1	TGGGAGGCCATCACATTGTA	TGTAATCCAGCAGGTCAGCAAA
IL-1α	TCATCCTGAATGACGCCCTC	TTATGTAATGCAGCAGCCGTG
IL-10	TGAAGACCCTCAGGCTGAGG	CACGGCCTTGCTCTTGTTTT
GRO-α	GTGTGAACGTGAAGTCCCCC	GCTTTCCGCCCATTCTTGA
ICAM-1	TGACGAAGCCAGAGGTCTCA	AGCGTCACCTTGGCTCTAGG
